# A single-nucleotide substitution of *CjTKPR1* determines pollen production in the gymnosperm plant *Cryptomeria japonica*

**DOI:** 10.1093/pnasnexus/pgad236

**Published:** 2023-08-08

**Authors:** Hiroyuki Kakui, Tokuko Ujino-Ihara, Yoichi Hasegawa, Eriko Tsurisaki, Norihiro Futamura, Junji Iwai, Yuumi Higuchi, Takeshi Fujino, Yutaka Suzuki, Masahiro Kasahara, Katsushi Yamaguchi, Shuji Shigenobu, Masahiro Otani, Masaru Nakano, Masaaki Nameta, Shinsuke Shibata, Saneyoshi Ueno, Yoshinari Moriguchi

**Affiliations:** Graduate School of Science and Technology, Niigata University, Niigata 950-2181, Japan; Institute for Sustainable Agro-ecosystem Services, Graduate School of Agricultural and Life Science, University of Tokyo, Tokyo 188-0002, Japan; Department of Forest Molecular Genetics and Biotechnology, Forestry and Forest Products Research Institute, Forest Research and Management Organization, Ibaraki 305-8687, Japan; Department of Forest Molecular Genetics and Biotechnology, Forestry and Forest Products Research Institute, Forest Research and Management Organization, Ibaraki 305-8687, Japan; Graduate School of Science and Technology, Niigata University, Niigata 950-2181, Japan; Department of Forest Molecular Genetics and Biotechnology, Forestry and Forest Products Research Institute, Forest Research and Management Organization, Ibaraki 305-8687, Japan; Forest and Forestry Technology Division, Niigata Prefectural Forest Research Institute, Niigata 958-0264, Japan; Forest and Forestry Technology Division, Niigata Prefectural Forest Research Institute, Niigata 958-0264, Japan; Graduate School of Frontier Sciences, The University of Tokyo, Chiba 277-8561, Japan; Graduate School of Frontier Sciences, The University of Tokyo, Chiba 277-8561, Japan; Graduate School of Frontier Sciences, The University of Tokyo, Chiba 277-8561, Japan; Trans-Scale Biology Center, National Institute for Basic Biology, Aichi 444-8585, Japan; Trans-Scale Biology Center, National Institute for Basic Biology, Aichi 444-8585, Japan; Faculty of Agriculture, Niigata University, Niigata 950-2181, Japan; Faculty of Agriculture, Niigata University, Niigata 950-2181, Japan; Graduate School of Medical and Dental Sciences, Niigata University, Niigata 951-8122, Japan; Graduate School of Medical and Dental Sciences, Niigata University, Niigata 951-8122, Japan; Department of Forest Molecular Genetics and Biotechnology, Forestry and Forest Products Research Institute, Forest Research and Management Organization, Ibaraki 305-8687, Japan; Faculty of Agriculture, Niigata University, Niigata 950-2181, Japan

**Keywords:** pollinosis, gymnosperm, conifer, pollen, male-sterility, Japanese cedar

## Abstract

Pollinosis, also known as pollen allergy or hay fever, is a global problem caused by pollen produced by various plant species. The wind-pollinated Japanese cedar (*Cryptomeria japonica*) is the largest contributor to severe pollinosis in Japan, where increasing proportions of people have been affected in recent decades. The *MALE STERILITY 4* (*MS4*) locus of Japanese cedar controls pollen production, and its homozygous mutants (*ms4/ms4*) show abnormal pollen development after the tetrad stage and produce no mature pollen. In this study, we narrowed down the *MS4* locus by fine mapping in Japanese cedar and found *TETRAKETIDE α-PYRONE REDUCTASE 1* (*TKPR1*) gene in this region. Transformation experiments using *Arabidopsis thaliana* showed that single-nucleotide substitution (“T” to “C” at 244-nt position) of *CjTKPR1* determines pollen production. Broad conservation of TKPR1 beyond plant division could lead to the creation of pollen-free plants not only for Japanese cedar but also for broader plant species.

**Significance Statement**

Pollinosis is a global health problem, but its genetic basis remains uncovered in pollinosis-caused plants. This study provides evidence that causal gene of *MALE STERILITY 4* (*MS4*) in Japanese cedar. The *TETRAKETIDE α-PYRONE REDUCTASE 1* (*TKPR1*) gene, encoding an enzyme that is essential for pollen wall construction, was identified as a causal gene of *MS4* and single-nucleotide substitution of *TKPR1* determines the pollen production. Conserved TKPR1 function among the broader plant species and succeeding of complementation with *TKPR1* of Japanese cedar in *Arabidopsis thaliana* indicate single-nucleotide substitution of *TKPR1* can generate pollen-free plants for broader plant species.

## Introduction

Pollen production is critical for plant reproduction; therefore, the genetics of pollen development have been studied extensively. This research has led to the discovery of the pollen developmental pathway, as well as many genes involved in pollen development regulation (1). Several gene mutants give rise to a pollen-free phenotype called male sterility (1, 2). In agriculture, pollen production is also important. Many pollen grains are required for artificial pollination and efficient seed/fruit production. Conversely, male sterility is a crucial trait required to produce hybrid crops with stable quality and quantity (3). For our health, pollen can sometimes be harmful by triggering an allergic reaction, pollinosis (4, 5).

Pollinosis is a global problem caused by pollen from various plant species, including both angiosperms and gymnosperms (4–11). For example, the prevalence of pollinosis is estimated at 40% in Europe (4). The global pollen load has increased in recent decades that thought mainly driven by climate change (12–14). Species of the family Cupressaceae, which belong to gymnosperms, are widely distributed over the eastern Mediterranean, Asia, and western North America. These species have long been used for timber or pharmaceutical products (e.g. essential oils, fragrances, and folk medicines); however, pollen produced by some Cupressaceae species are major triggers of severe pollinosis; these include Arizona cypress (*Cupressus arizonica*), Italian cypress (*Cupressus sempervirens*), mountain cedar (*Juniperus ashei*), Japanese cypress (*Chamaecyparis obtusa*), and Japanese cedar (*Cryptomeria japonica*) (10, 15).

**Fig. 1.**
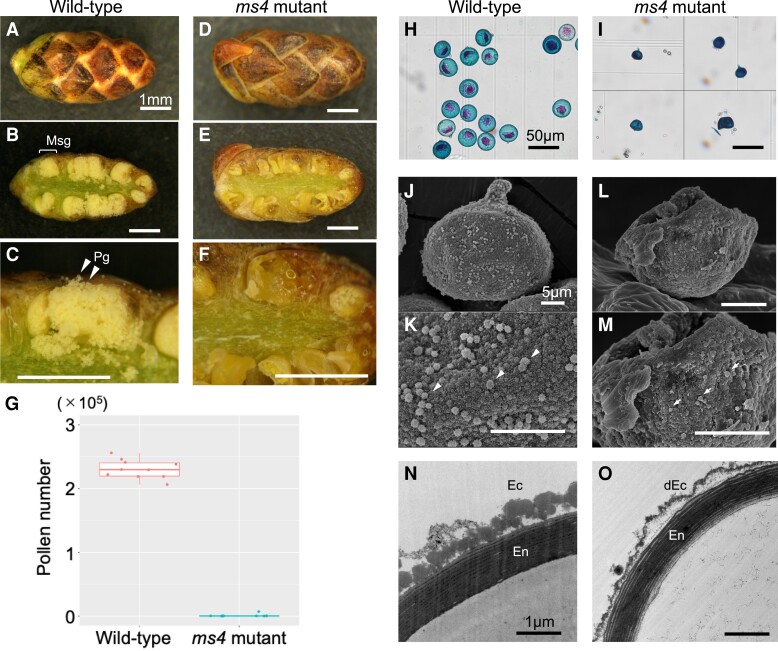
Male strobili and pollen from WT and *ms4* mutant Japanese cedar plants. A–F) Male strobili of the WT (“Higashikanbara-5”; A–C) and *ms4* mutant (“Shindai-8”; D–F). Scale bars, 1 mm. Transverse-cut sample from the WT (B and C) showed many pollen grains (Pg) in each microsporangium (Msg), whereas the *ms4* mutant had no or a few pollen grains in microsporangia (E and F). G) Pollen grain count for a single male strobilus. Although approximately 200,000 pollen grains were produced in the WT, a few pollen grains (median = 0–7,000) were counted in the *ms4* mutant (*n* = 10, 10). H and I) Pollen grain observations following Alexander staining. WT plants produced globular pollen grains with single projections (papillae) (H), whereas *ms4* plants produced small, squashed pollen grains. Scale bars, 50 *μ*m. J–M) SEM observations of normal (J and K) and *ms4* mutant (L and M) pollen. Although normal pollen had uniform orbicules (arrows, K), *ms4* mutant pollen had small, irregular orbicules (arrows, M). Scale bars, 5 *μ*m. N and O) Pollen section observation of normal (N) and *ms4* mutant pollen (O) by TEM. No clear differences were observed for endexine (inner pollen wall structure, “En” in N and O) from normal and *ms4* pollen. However, degenerated ectexine structure was observed in *ms4* pollen (“dEc” in O) compared with normal pollen (“Ec” in N). Scale bars, 1 *μ*m. Data shown in G) are provided in a source data file.

Japanese cedar is a wind-pollinated conifer and is the most important timber species in Japan, accounting for 58% (12.7 million m^3^) of total roundwood production in 2019 (16). However, Japanese cedar is also the leading cause of the most severe allergic reactions to pollen in Japan (11). A single Japanese cedar tree typically produces thousands of male strobili, which correspond to male flowers in angiosperms; a single male strobilus contains approximately 100,000–300,000 pollen grains (Fig. 1A–C; Movie S1) (17). Upon release, these vast numbers of pollen grains are carried away by the wind. The proportion of people suffering from pollinosis caused by Japanese cedar pollen in Japan has increased in recent years, from 16.2% in 1998 to 26.5% in 2008 and 38.8% in 2019 (18, 19). Therefore, planting pollen-free trees is a promising strategy for maintaining Japanese cedar as a building material while countering its effects in pollinosis. According to these situations and demand, several pollen-free cultivars were bred (20), and the annual production of seedlings of low-pollen and pollen-free Japanese cedar has been increasing yearly in Japan (21). Unveiling the genetic mechanism of pollen-free mutants will contribute not only to promoting pollen-free timber breeding but also to understanding the flowering mechanism in gymnosperm plants.

Conifers have large genomes, which complicates genetic studies of these species (e.g. 25.4 Gb for *Pinus tabuliformis* (22) and ∼20 Gb for *Picea abies* (23)). Flowering trait analyses are particularly problematic due to the necessity of large fields for keeping materials for segregation analyses, as well as a wait time of several years until the flowering stage can be examined (24). Among conifers, Japanese cedar has several advantages for flowering trait analyses. Its genome is smaller (∼10.8 Gb) (25) than those of other conifer species, and its flowering can be induced artificially using gibberellic acid (26). Previous field studies identified four male sterility loci, i.e. *MALE STERILITY 1–4* (*MS1–MS4*), in Japanese cedar (27, 28), through linkage analyses (28, 29). Recently, a strong candidate gene for *MS1* was discovered by our research (30). Two different mutation alleles of *CJt020762*, which are located on the *MS1* locus, showed pollen-free phenotype, suggesting that *CJt020762* is a causal gene of *MS1*, although functional validation is required. Here, we focus on identifying the causal gene in the *MS4* locus. We find abnormal pollen wall structure at the microspore stage in the *ms4* mutant. Fine-mapping and RNA-sequencing (RNA-seq) analysis suggest the *CjTKPR1* gene is the strong candidate gene for the causal gene of *MS4*. Furthermore, functional validation with *Arabidopsis thaliana* reveals single-nucleotide substitution of *CjTKPR1* determines pollen production.

## Results

### Male strobilus and pollen observation of *ms4* mutant

The *ms4* mutant “Shindai-8” was discovered in a planted forest in Niigata Prefecture, Japan. Mature male strobili of homozygous *ms4* mutants (*ms4/ms4*) produced almost no pollen (Fig. 1A–G). Very little of the pollen produced by the *ms4* mutants showed obvious abnormal phenotypes compared to wild-type (WT) plants, such as small or collapsed pollen grains (Fig. 1H and I). Scanning electron microscope (SEM) observations revealed that the *ms4* mutant produces small, abnormal orbicules, which are components of pollen wall (Fig. 1J–M) (31). Furthermore, transmission electron microscope (TEM) observation a showed thick ectexine structure of pollen wall in WT as previously reported (“Ec” in Fig. 1N (32)). On the other hand, *ms4* mutant pollen had degenerated ectexine (“dEc” in Fig. 1O). Pollen wall is an essential constituent of the pollen, and dysfunctional biosynthesis genes often result in male sterility (33, 34).

### Fine mapping of *MS4* locus and transcriptome analysis

**Fig. 2.**
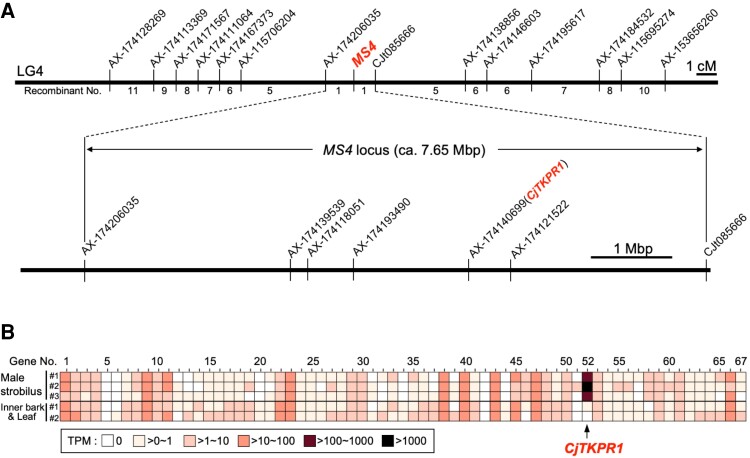
Fine mapping and analysis of candidate genes in the *MS4* locus. A) Fine mapping of the *MS4* locus according to a linkage map around *MS4* (top) and physical map of the *MS4* region (bottom). B) Expression patterns of 67 genes of the *MS4* region from male strobili (three samples) or inner bark and leaf tissues (two samples). RNA-seq data were obtained from our previous study (35) and expression levels were categorized into six levels (0, >0–1, >1–10, >10–100, >100–1,000, or >1,000 TPM). Gene no. 52, *CjTKPR1*, had the highest expression level among male strobilus samples but no or little expression was detected in inner bark or leaf tissues. Precise expression data for each gene are provided in Table S3.

To narrow down the region of *MS4* locus, we generated a segregating population by crossing the *ms4* mutant with heterozygous individuals (“Sindai-8” × “S8HK5”; Table S1). We found that 46 individuals produced normal pollen, whereas 48 showed pollen-free phenotype (Table S2). Then, we conducted fine mapping using these 94 individuals and narrowed down *MS4* for an approximately 7.65-Mb-wide region on linkage group 4 (Fig. 2A). Annotation analysis revealed 67 genes in this region (Fig. 2B and Table S3). Among these genes, we found a homologous gene of *TETRAKETIDE α-PYRONE REDUCTASE 1* (*TKPR1*) (hereafter *CjTKPR1*); this gene is essential for sporopollenin biosynthesis through making the exine layer of the pollen wall (33, 36). RNA-seq data revealed that *CjTKPR1* had the highest expression levels in all three male strobilus samples (183.17–1,131.88 transcripts per million [TPM]), but less or no expression in inner bark and leaf tissues (0 and 0.54 TPM, respectively; Fig. 2B and Table S3) (35).

### Male organ-specific expression and sequence comparison of *TKPR1* suggest that *TKPR1* is causal gene of *MS4*

**Fig. 3.**
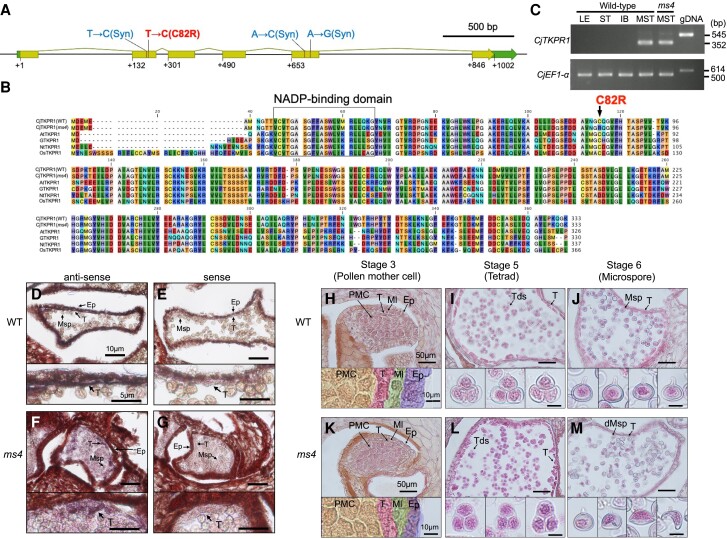
Analysis of *CjTKPR1*. A) Gene structure of *CjTKPR1*. Outer/green and inner/yellow boxes indicate mRNA and coding regions, respectively. Four nucleotide substitutions between WT and *ms4* mutant (“Shindai-8”) are shown above the boxes. Only a single amino acid substitution was detected at position 244 nt (C82R); the others were synonymous mutations (Syn). B) Amino acid comparison among CjTKPR1, AtTKPR1, GTKPR1, NtTKPR1, and OsTKPR1. A single amino acid substitution of *ms4* mutant (C82R, arrow) was conserved within various plant species (Fig. S1). The putative NADP-binding domain is framed. Multiple-sequence alignment was generated using CLC Main Workbench v22.0 software. C) RT-PCR of the *CjTKPR1* transcript. *ELONGATION FACTOR1-alpha* (*CjEF1-α*) was used as an internal control. gDNA, genomic DNA; IB, inner bark; LE, leaf; MST, male strobilus; ST, stem. D–G) In situ hybridization of *CjTKPR1*. Magnified views are shown at the bottom of each figure. *CjTKPR1* expression was observed in the tapetum (T) of both WT (D) and *ms4* mutant plants (F), whereas no signal was observed from the sense probe (E and G). Scale bars: top = 10 *μ*m, bottom = 5 *μ*m. H–M) Transverse section of male strobilus development in WT (H–J) and *ms4* mutant plants (K–M). Microsporangia of male strobili from stages 3 (H and K), 5 (I and L), and 6 (J and M). Magnified views are shown at the bottom of each figure. Male strobilus stages were defined previously (41). dMsp, degraded microspores; Ep, epidermis; Ml, middle layer; Msp, microspore; PMC, pollen mother cell; T, tapetum; Tds, tetrads. Scale bars: top = 50 *μ*m, bottom = 10 *μ*m.

*TKPR1* is expressed in the tapetum, and tetraketide reductase activity of TKPR1 was observed in vitro for *A. thaliana* and tobacco (37, 38). Mutants of *TKPR1* lead to male sterility in several angiosperm species such as *A. thaliana*, rice, tobacco, and gerbera (36–39). Histological observations have revealed that mutants of *AtTKPR1* and *OsTKPR1* show no obvious defects at the tetrad stage, although abnormal microspores appeared in later stages and pollen cells, before degenerating rapidly (39, 40). CjTKPR1 shares 54.6–64.9% amino acid identity with *A. thaliana*, gerbera, tobacco, and rice (Figs. 3A and B and S1). Reverse-transcription polymerase chain reaction (RT-PCR) showed specific *CjTKPR1* expression in the male strobilus from WT and *ms4* mutant (Fig. 3C). Furthermore, in situ hybridization of male strobili confirmed *CjTKPR1* expression in the tapetum of Japanese cedar (Fig. 3D–G). Histological observations of male strobili showed that the *ms4* mutant not only has a clear tetrad structure but also has crushed, abnormal microspores (Fig. 3H–M) as previously observed in *A. thaliana* and rice (39, 40). These results support the hypothesis that *CjTKPR1* is the causal gene of *MS4* in Japanese cedar.

Sequence comparison of *CjTKPR1* between WT (“Higashikanbara-5”) and *ms4* (“Shindai-8”) revealed only four nucleic acid substitutions; interestingly, only one amino acid substitution was detected at position 244 nt (C82R; Fig. 3A and B) among these substitutions. This single-nucleotide polymorphism (SNP) at 244 nt was completely linked with the pollen-free phenotype in wild accessions (Table S1) and two crossing progenies (“S8” × “S8HK5,” Table S2; selfing progenies of “S8DY1,” Table S4), except for mutants from other male sterility loci (*ms1–ms3*). Thus, only plants with C/C at position 244 nt showed the pollen-free phenotype, and those having T/T or T/C showed normal pollen production (Tables S1, S2, and S4). This cysteine-82 of CjTKPR1 was perfectly conserved in various plant species, including both angiosperms and gymnosperms (Figs. 3B and S1). Furthermore, evaluation tools for amino acid substitution predicted that C82R leads to severe defects for CjTKPR1 (Fig. S2 (42, 43)). These results suggest that *CjTKPR1* is an important gene for pollen production in Japanese cedar and that the nucleotide substitution at position 244 nt (C82R) is a strong candidate for the causal mutation of *MS4* in Japanese cedar.

### Functional analysis of *CjTKPR1* using *A. thaliana*

**Fig. 4.**
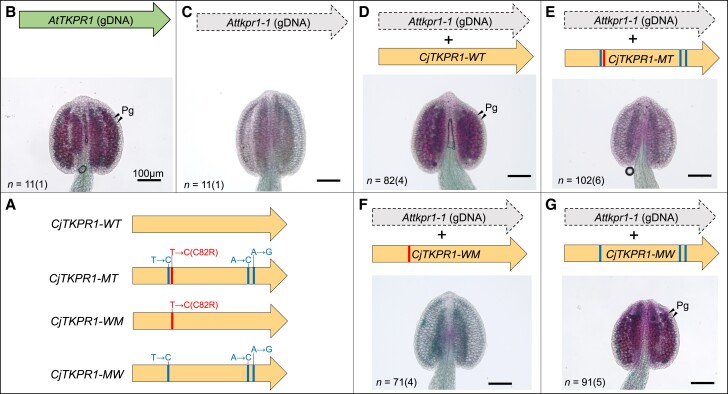
Complementation analysis of *CjTKPR1*. Four types of *CjTKPR1* sequences were constructed (A) and transgene mutants were prepared in the *Attkpr1-1/Attkpr1-1* background. “T→C(C82R)” (also shown as red line) and “T→C”, “A→C”, “A→G” (also shown as blue lines) indicate *ms4* SNPs, where “T→C(C82R)” indicates a single-amino acid substitution (C82R, 244-nt position) and the other three lines indicate synonymous mutation. The *Attkpr1-1* homozygous mutant showed pollen-free phenotype in contrast to the WT (B and C). Plants inserted with *CjTKPR1-WT* and *CjTKPR1-MW* (with T at 244 nt) recovered their pollen production (D and G), whereas those inserted with *CjTKPR1-MT* and *CjTKPR1-WM* (with C at 244 nt) showed no pollen production (E and F). Expressions of the inserted *CjTKPR1* were confirmed by RT-PCR (Fig. S3). Numbers indicate anther counts, followed by the number of biological replicates in parentheses. Data shown in B)–G) are provided in a source data file. Pg: Pollen grain.

We conducted complementation tests to confirm that *CjTKPR1* sequences control pollen production using *A. thaliana* (Fig. 4). First, we obtained the *TKPR1* mutant of *A. thaliana* (*Attkpr1-1* mutant, SAIL_837_D01), which homozygous plant shows a pollen-free phenotype (Fig. 4B and C) (37). Then, four *CjTKPR1* sequences, i.e. the WT, *ms4* mutant (MT), WT background with a point mutation at 244 nt (T to C, WM), and *ms4* background with a point mutation at 244 nt (C to T, MW) (Fig. 4A), were each introduced into heterozygous plants of *AtTKPR1* (*AtTKPR1/Attkpr1-1*), not into *Attkpr1-1* homozygous plants (*Attkpr1-1/Attkpr1-1*) that produce no pollen and therefore obtain no progenies. Next, we selected *Attkpr1-1* homozygous plants with *CjTKPR1* constructs from the T2 or T3 generations. Anthers of these plants were stained by Alexander staining and pollen production was confirmed (44). As a result, *CjTKPR1-WT*-introduced plants recovered pollen production (Fig. 4D), whereas *CjTKPR1-MT*-introduced plants still showed the pollen-free phenotype (Fig. 4E). These results indicate that *CjTKPR1-WT* can complement pollen production in *A. thaliana* and that the *CjTKPR1* allelic difference controls pollen production. Lines with a single-nucleotide substitution at 244 nt showed that *CjTKPR1-WM*-introduced plants, which have a *CjTKPR1-WT* background with a point mutation at 244 nt, could not recover pollen production (Fig. 4F). However, *CjTKPR1-MW*-introduced plants, which have a *ms4* background without a point mutation at 244 nt, recovered pollen production (Fig. 4G). Together, these complementation test results indicate that single-nucleotide substitution of *CjTKPR1* at 244 nt determines pollen production.

## Discussion

In this study, we identified a single-nucleotide substitution of *CjTKPR1* as a causal mutation of *MS4* in Japanese cedar. This knowledge provides the basic information for selecting and breeding pollen-free Japanese cedar efficiently and precisely. Our results also contribute for the creation of new pollen-free plants using genome editing by targeting *TKPR1* gene. Cupressaceae species are suitable target plant species because they are often used for timber but are also global sources of severe pollinosis including Arizona cypress, Italian cypress, and mountain cedar (15). Since the recent progress of genome-editing methods that enable the application of mutagenesis for broader plant species, including Japanese cedar (45), combining our results with genome editing methods will accelerate the creation of new pollen-free plants.

Tapetum expression of *CjTKPR1* in both WT and *ms4* mutant plants suggests that *CjTKPR1* of *ms4* may be dysfunctional at the protein level, but not at the transcription level, for example in terms of tetraketide reductase activity (Fig. 3D–G). This study identified the cause of dysfunction of TKPR1 activity at the single amino acid level. Because the activity domain of TKPR1 has not yet been analyzed well, our results give a new insight into the enzymatically important amino residues for tetraketide reductase activity of TKPR1.

Interestingly, TKPR1 occurs not only in seed plants but also in phylogenetically basal plants without pollen, such as the fern *Ceratopteris richardii* and liverwort *Marchantia polymorpha* (Fig. S1). Although ferns and liverworts produce sperm as male gametes and do not have a pollen wall structure, they have a perispore layer within the spore that consists of sporopollenin. Sporopollenin is thought to be a key structure for the colonization of terrestrial environments during plant evolution, protecting them from ultraviolet B light (46, 47). Our results that *CjTKPR1* could rescue dysfunction of *TKPR1* of *A. thaliana* may imply that other pollen developmental genes are also conserved among the Plantae. Further investigation of the pollen developmental genes of Japanese cedar and comparison thereof among other plant species will help elucidate the evolution of male gametes in plants.

## Materials and methods

### Plant materials

Male strobili of Japanese cedar were collected from planted fields. We used 10 lines of wild accessions, three crossing parents (Table S1), and two combinations of crossing progenies (Tables S2 and S4). Detailed information including sample name, sampling location, pollen phenotype, and SNP types at 244 nt of *CjTKPR1* for all the Japanese cedar materials used in this study is listed in Tables S1, S2, and S4. DNA and RNA were extracted from needle (29) and male strobilus (30) tissues, as described previously.

### Male strobilus and pollen observations

For the male strobilus observations, WT (“Higashikanbara-5”) and *ms4* mutant (“Shindai-8”) of Japanese cedar plants were used. Male strobili were cut using a razor and photographed under a stereomicroscope (SZ-11; Olympus, Tokyo, Japan) using a digital camera (Wraycam EL310; Wraymer, Osaka, Japan). Pollen grains were stained with Alexander staining solution (38) and photographed under a light microscope (BX-50; Olympus). Pollen grains from “Higashikanbara-5” and “Shindai-8” were counted using a cell counter (48). Briefly, a single male strobilus was gently squashed using a pestle and suspended with water. The pollen suspension was then filtrated through two mesh filters (100 and 20 *μ*m) to remove male strobilus debris and dust. The cleaned suspension was mixed with a cell counter buffer (CASYton; OLS, Bremen, Germany), and pollen grains were counted using a CASY cell counter (OLS).

### SEM

Pollen grains were extracted from progenies of “S8” × “S8HK5” (“P411” as normal pollen and “P380” as *ms4* mutant; Table S1). Male strobili were harvested on 2019 December 12, after pollen maturation but before pollen release. After washing with deionized water, the male strobili were air-dried for 2 days. Pollen was harvested from dried male strobili and further sifted through a 75-*μ*m mesh. The samples were coated using an osmium plasma ion coater (OPC80N; Filgen, Nagoya, Japan) and SEM observations were performed using a JSM-7500F system (JEOL, Tokyo, Japan) at 5 kV.

### TEM

The male strobili in FAA solution (formalin:acetic acid:70% ethanol = 5:5:90) were sectioned into eight parts and washed with 0.1 m phosphate buffer (pH 7.4). Then, the male strobilus was fixed in 4% paraformaldehyde in 0.1 m phosphate buffer (pH 7.4) for 48 h (decompression using a syringe for the first 3 h) at room temperature and then fixed in 2.5% glutaraldehyde in 0.1 m phosphate buffer (pH 7.4) for 3 h at 4°C. Following a wash with 0.1 m phosphate buffer (pH 7.4), the strobilus was further postfixed in 2% osmium tetroxide in 0.1 m phosphate buffer (pH 7.4) for 2 h at room temperature and then rinsed with distilled water. The male strobilus was dehydrated using graded concentration of ethanol solutions and immersed in propylene oxide twice for 1 h each. To replace the epoxy resin (Quetol651, Nissin EM, Tokyo, Japan), the male strobilus was embedded in the resin with propylene oxide. Specifically, they were immersed for 2 h in 1/3 Quetol, 2 h in 2/3 Quetol, and 1 h in 3/3 Quetol, and then they were left to stand for 4 days while being replaced with 3/3 Quetol once a day (decompression using a syringe only on the first day) and were polymerized at 60°C for 48 h. Ultrathin sections (75 nm) were obtained using an Ultramicrotome (EM UC7; Leica Microsystems, Wetzlar, Germany) and stained with 2% uranyl acetate and 1% lead citrate. The samples were observed under TEM (JEM-1400; JEOL) at an accelerating voltage of 80 kV.

### Fine mapping of the *MS4* locus

Linkage maps were constructed using a *MS4* mapping family consisting of 46 normal pollen production offspring and 48 male sterile offspring produced by artificial crossing (“S8” × “S8HK5”; Table S2). The SNP marker AX-174140699 on *CjTKPR1* was previously mapped on LG4 with five adjacent markers (29). These markers were developed from transcript sequences blasted against two versions (0.11 and 0.1) of draft genome assemblies of *C. japonica*, leading to the identification of three genomic contig sequences (ctg370 and ctg2011 from ver. 0.11 and ctg482 from ver. 0.1). These three genomic contig sequences were manually combined and genomic coordinates were calculated, starting from AX-174206035 (Fig. 2A; Table S3). Transcript sequences from *C. japonica* (CJ3006NRE) (35) were mapped against genomic contig sequences using the minimap2 tool and the “-ax splice” option, resulting in 67 transcripts (Table S3). An additional marker (CJt085666; Table S3) was genotyped for the mapping family using PCR–restriction fragment length polymorphism (RFLP) and the *Nde*I restriction enzyme, to obtain *CjTKPR1* and the genetic markers on either side (Fig. 2A).

### RT-PCR

cDNA was synthesized from 500-ng aliquots of total RNA from Japanese cedar and *A. thaliana* using the PrimeScript IV 1st Strand cDNA synthesis kit (Takara Bio, Shiga, Japan). *CjTKPR1* and *EF1-α* were amplified using intron-containing primers to distinguish between cDNA and gDNA amplification. The primer sequences are listed in Table S5.

### In situ hybridization

Full-length cDNA of *CjTKPR1* was cloned into the pZErO vector (Invitrogen, Waltham, MA, USA). Subsequently, 150-bp DNA fragments were amplified from the cloned full-length cDNA using gene-specific primers containing SP6 or T7 RNA polymerase promoter sequences at the 5′-end (Table S5). PCR products were purified through phenol/chloroform/isoamyl alcohol extraction followed by ethanol precipitation and used as templates for in vitro transcription. Sense and anti-sense digoxigenin-labeled probes were synthesized using the DIG RNA Labeling Kit (SP6/T7; Roche, Basel, Switzerland) according to the manufacturer's instructions.

Male strobili of WT (“Nakakubiki-4”) and *ms4* mutant (“P380”) plants were fixed in FAA solution (formaldehyde:acetic acid:70% ethanol = 5:5:90) at 4°C for 24 h. After dehydration and embedding, the strobili were cut to a thickness of 20 *μ*m. Hybridization and immunological detection were performed as described previously (49, 50).

### Histological analysis of male strobili

Male strobili were collected from “Santo-1” (WT) and “Shindai-8” (*ms4* mutant) plants, which were grown at the Niigata Prefectural Forest Research Institute. Male strobili were fixed with FAA, dehydrated using graded ethanol series, introduced to chloroform through a chloroform–ethanol series, and embedded in paraffin. We cut 10-*μ*m sections using a microtome (ESM-100L; ERMA, Tokyo, Japan), which were then stained with Mayer's hematoxylin solution and mounted in Eukitt mounting medium (ORSAtec, Bobingen, Germany). These sections were observed under a light microscope (BX-50; Olympus) equipped with a digital camera (Wraycam-NOA2000; Wraymer).

### Transgenic experiments

Total RNA of *CjTKPR1* was extracted from male strobili of *Ms4/ms4* heterozygous individual (“S8TM4”; Table S1). cDNA was synthesized using the PrimeScript II kit (Takara Bio). Total RNA and genomic DNA from *A. thaliana* (Col-0 accession) were extracted from flower buds and leaf tissues, respectively. Coding regions of *CjTKPR1* and *AtTKPR1* were amplified using PrimeStar GXL polymerase (Takara Bio) with specific primers (Table S5). pENTR1a, *AtTKPR1* promoter (pro), coding sequences of *CjTKPR1* or *AtTKPR1*, and Nos terminator (NosT) fragments were combined with In-Fusion HD mix (Takara Bio) and cloned to *Escherichia coli*. Single-nucleotide substitution constructs were made by inverse PCR with mutation-containing primers (Table S5). Each AtTKPR1pro:TKPR1 coding sequence:NosT construct was further cloned into a pFAST-G01 vector using Gateway LR clonase II (Thermo Fisher Scientific, Waltham, MA, USA). We prepared *AtTKPR1/Attkpr1-1* heterozygous plants (Col-0 background; accession no. CS837358) as the transformation hosts because the *Attkpr1-1* homozygous mutant produces no pollen and therefore no progenies. Each construct was transformed into *AtTKPR1* heterozygous mutants via *Agrobacterium tumefaciens* (GV3101) using the floral dip method (51). *AtTKPR1* heterozygous individuals containing an external *TKPR1* construct were selected based on fluorescence in the seed and confirmed by PCR (Table S5). For the next generation, we selected *Attkpr1-1* homozygous mutants with external *TKPR1* sequences. Pollen production was analyzed based on anther observation under a microscope after Alexander staining (44).

## Supplementary Material

pgad236_Supplementary_Data

## Data Availability

The genomic DNA sequence of *CjTKPR1* from “Shindai-8” (*ms4/ms4*) has been deposited in the DNA Data Bank of Japan (accession no. LC773408). All data needed to evaluate the conclusions in the paper are present in the paper and/or the Supplementary material.
