# Supplementary material for: A single-nucleotide substitution of CjTKPR1 determines pollen production in the gymnosperm plant Cryptomeria japonica
Source: PNAS Nexus. 2023 Aug 8;2(8):pgad236. doi: 10.1093/pnasnexus/pgad236 (PMC10408704; doi:10.1093/pnasnexus/pgad236)
Supplement: pgad236_Supplementary_Data [file pgad236_supplementary_data.zip › PNASNEXUS-PNASNEXUS-2022-01010R-s01.docx]

**
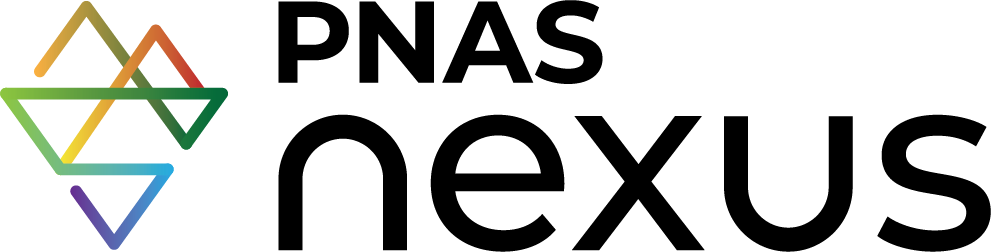
**

**Supplementary Information for**

A single-nucleotide substitution of *CjTKPR1* determines pollen production in the gymnosperm plant *Cryptomeria japonica*

Hiroyuki Kakui^a,b,1^, Tokuko Ujino-Ihara^c,1^, Yoichi Hasegawa^c^, Eriko Tsurisaki^a^, Norihiro Futamura^c^, Junji Iwai^d^, Yuumi Higuchi^d^, Takeshi Fujino^e^, Yutaka Suzuki^e^, Masahiro Kasahara^e^, Katsushi Yamaguchi^f^, Shuji Shigenobu^f^, Masahiro Otani^g^, Masaru Nakano^g^, Masaaki Nameta^h^, Shinsuke Shibata^h^, Saneyoshi Ueno^c,^*, Yoshinari Moriguchi^g,^*

**Author affiliations**

^a^Graduate School of Science and Technology, Niigata University, Niigata, 950-2181, Japan.

^b^Present address: Institute for Sustainable Agro-ecosystem Services, Graduate School of Agricultural and Life Science, University of Tokyo, 188-0002, Tokyo, Japan.

^c^Department of Forest Molecular Genetics and Biotechnology, Forestry and Forest Products Research Institute, Forest Research and Management Organization, 305-8687, Ibaraki, Japan.

^d^Niigata Prefectural Forest Research Institute, 958-0264, Niigata, Japan.

^e^Graduate School of Frontier Sciences, The University of Tokyo, 277-8561, Chiba, Japan.

^f^Trans-Scale Biology Center, National Institute for Basic Biology, 444-8585, Aichi, Japan.

^g^Faculty of Agriculture, Niigata University, 950-2181, Niigata, Japan.

^h^Graduate School of Medical and Dental Sciences, Niigata University, 951-8122, Niigata, Japan

^1^These authors contributed equally to this work.

*Corresponding authors: Saneyoshi Ueno, Yoshinari Moriguchi

**Email:**  saueno@ffpri.affrc.go.jp (S.U.); chimori@agr.niigata-u.ac.jp (Y.M.)

**This PDF file includes:**

Supplementary text

Figures S1 to S2

Tables S1 to S5

Legends for Movies S1

Legends for Datasets S1 to S6

**Other supplementary materials for this manuscript include the following:**

Movies S1

Datasets S1 to S6

(a)

**
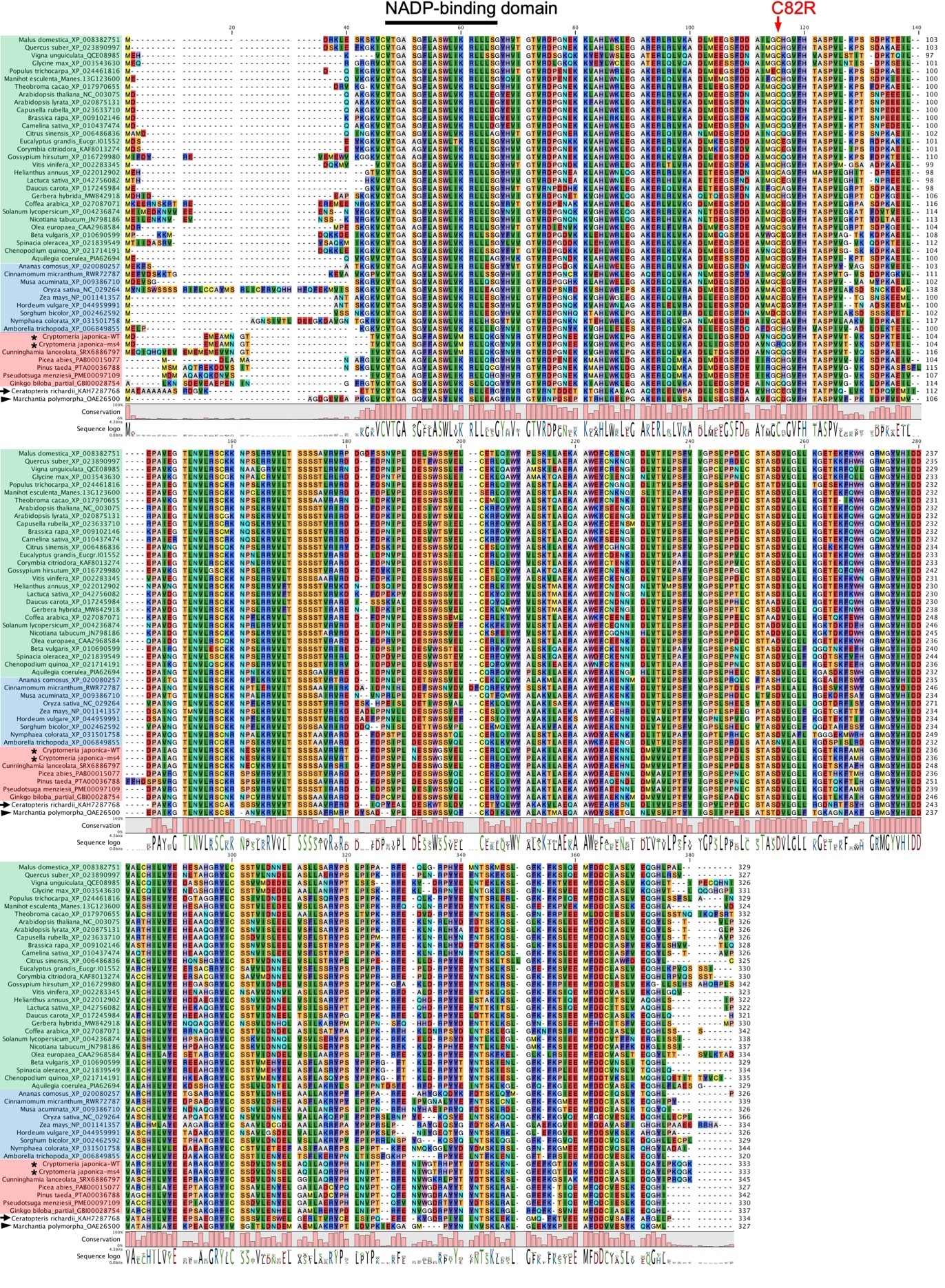
**

(b)

**Fig. S1** Comparison of TKPR1 protein sequences from various plant species including angiosperms, gymnosperms, and phylogenetically basal plant species. (a) Alignment of TKPR1. All TKPR1 sequences had a conserved cysteine at the 82^nd^ residue of CjTKPR1 (red arrow), except for the *ms4* mutant. Eudicots and monocots (angiosperms) are highlighted in green and blue, respectively. Gymnosperms are highlighted in red. Asterisks indicate Japanese cedar. Black arrow and arrowhead indicate *Ceratopteris richardii* and *Marchantia polymorpha*, which are phylogenetically basal plant species that do not produce pollen. The putative NADP-binding domain is outlined. (b) Amino acid identity and differences. Red boxes indicate comparison with CjTKPR1. Accession numbers are provided next to the species name. Multiple-sequence alignment was generated using CLC Main Workbench v22.0 software.

**(a)**

**
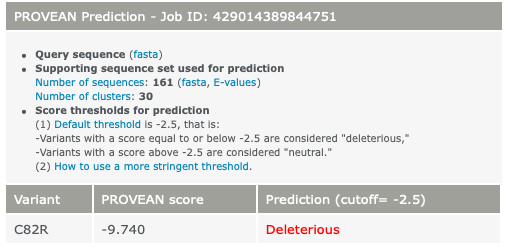
**

**(b)**


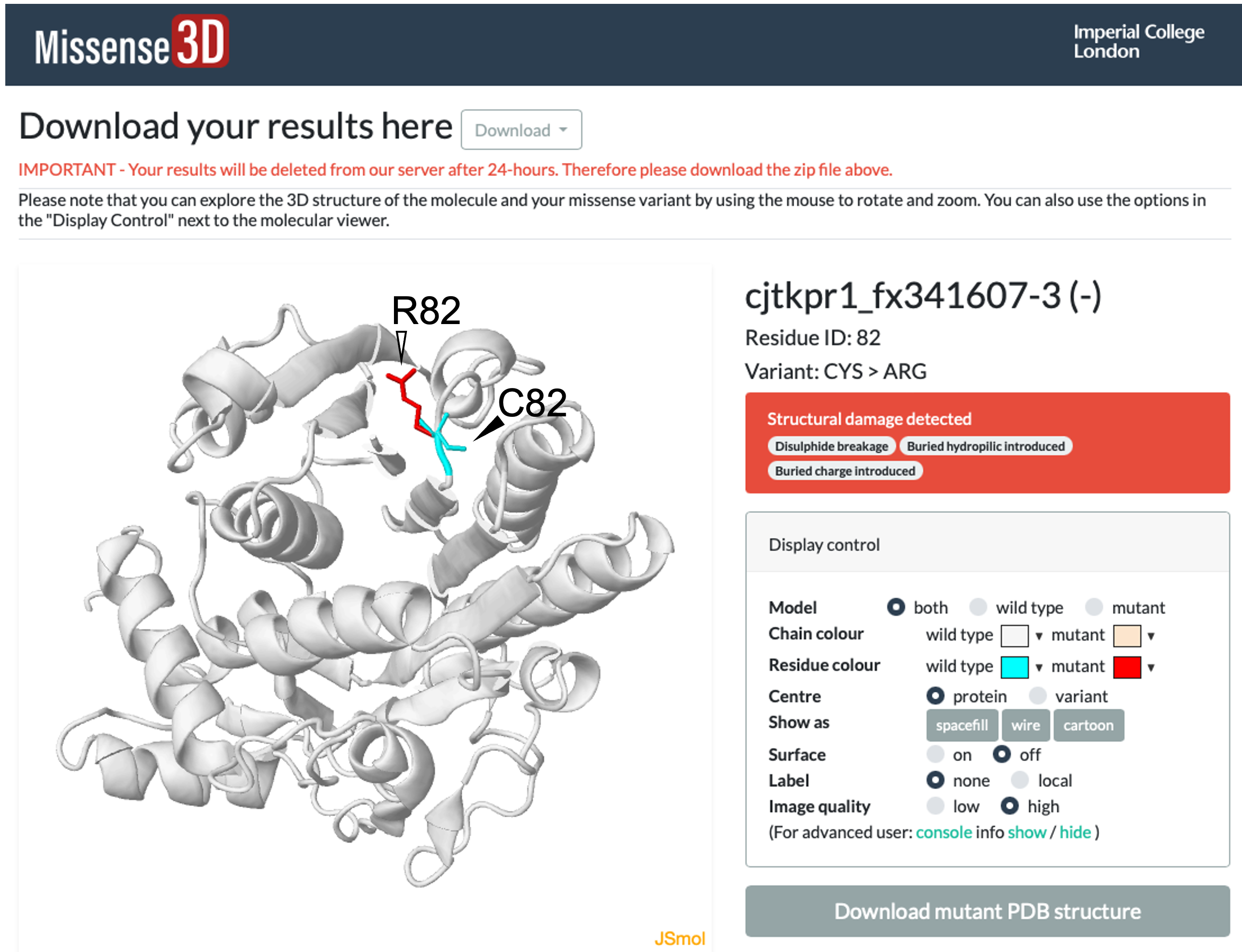

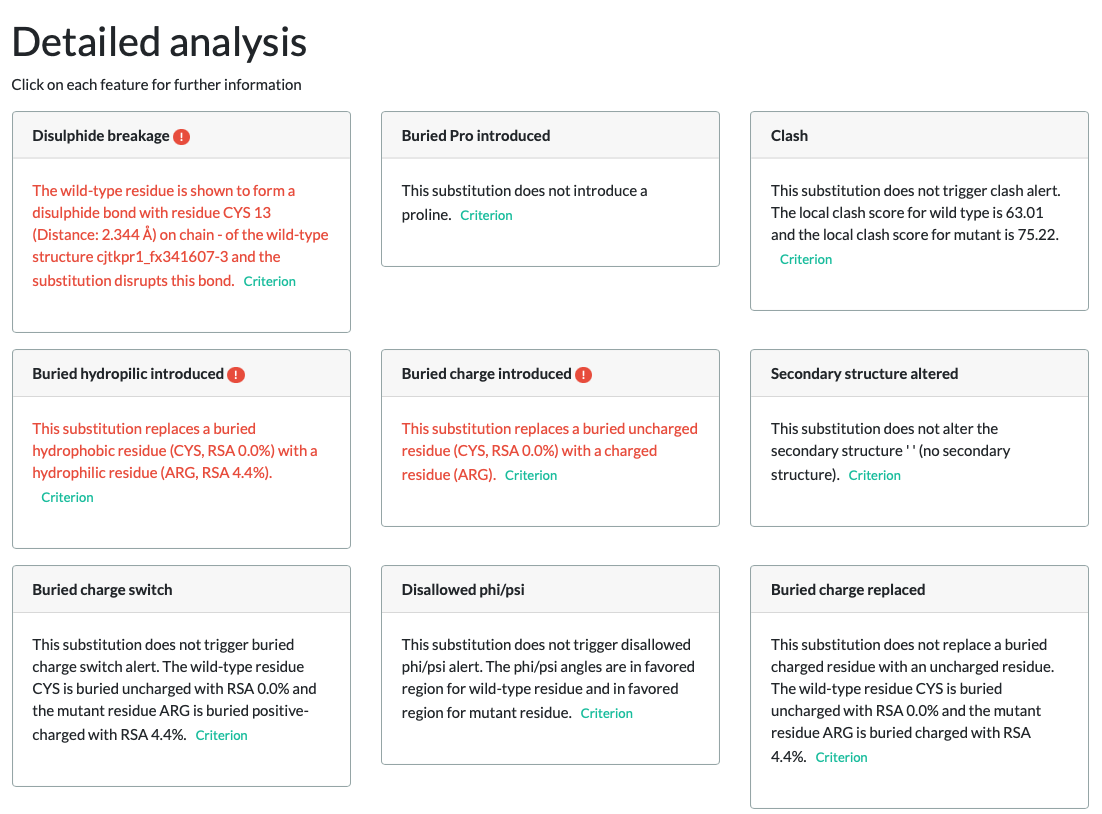

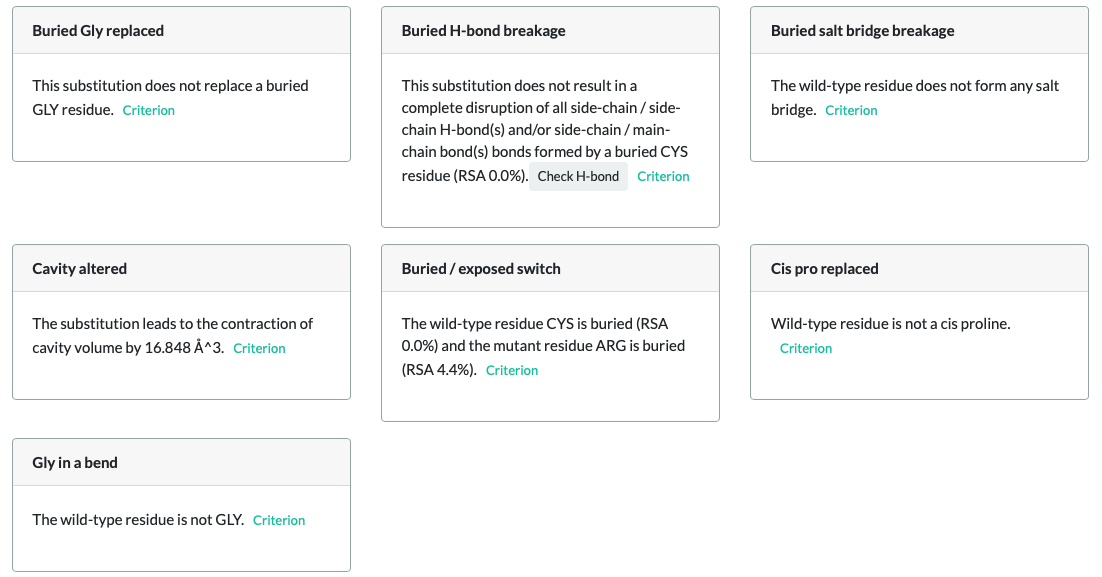


**Figure S2 (a)** PROVEAN, the web tool for the evaluation of the amino acid substitution, predicted C82R of CjTKPR1 is a deleterious substitution (42). **(b)** Structural changes, including disulfide bond breakage (C82: filled triangle, R82: empty triangle), were predicted by Missense3D (43).

**Fig. S3** *CjTKPR1* expression of complementation mutants in *A. thaliana*. The *ELONGATION FACTOR 1-alpha* (*AtEF1-α*) gene was used as an internal control (a). Expression in each *TKPR1* mutant was confirmed by RT-PCR (b) and RT-PCR-RFLP (c). CjWT, *CjTKPR1*-*WT*; CjWM, *CjTKPR1*-*WM*; CjMT, *CjTKPR1-MT*; CjMW, *CjTKPR1-MW* (Fig. 4A). Schematic structures of *AtEF1-α* (d) and *CjTKPR1* (e). Primer information is provided in Table S5.

Table S1. Information of Japanese cedar materials in this study

**Table S2. Pollen production and SNP genotype for progenies of S8 (*ms4/ms4* ) × S8HK5 (*Ms4/ms4* )**

Each individual data is provided as a Source Data file

**Table S3. Annotated sixty seven genes in the *MS4* locus**

RNA-seq data were obtaind from Wei *et al*. 2021 (35).

**Table S4. Pollen production and SNP genotype for selfed progenies of S8DY1(*Ms4/ms4* )**

Each individual data is provided as a Source Data file

**Table S5. Primer list in this study**

**Legends for separate files**

**Movie S1 Time-lapse movie of flowering Japanese cedar male strobili.** Male strobili are initially closed (0–8 s). Then, the strobili begin to open, and microsporangia (yellow spherical structures) appear (8–14 s). Finally, microsporangia break open and pollen grains are released (14–23 s). The video was recorded using a TLC200 Pro camera (Brinno, Taipei, Taiwan) from 7th to 10th, May 2020. Time points are indicated at the bottom of the video frame in the following format: yyyy/mm/dd, hh:mm:ss.

**Dataset S1** **for Fig.1G.** Pollen grain number of wild-type (Higashikanbara-5) and *ms4* mutant (Shindai-8).

**Dataset S2 for Fig. 2A.** Genotype list (all).

**Dataset S3 for Fig. 2A.** Genotype list (around *MS4* locus).

**Dataset S4 for Fig. 4.** Detailed information for complementation tests.

**Dataset S5 for Table S2.** Pollen phenotype and SNP genotype for progenies of S8 (*ms4/ms4*) × S8HK5 (*Ms4/ms4*).

**Dataset S6 for Table S4.** Pollen phenotype and SNP genotype for selfed progenies of S8DY1(*Ms4/ms4*).
